# Changes in the Fruit Quality, Phenolic Compounds, and Antioxidant Potential of Red-Fleshed Kiwifruit during Postharvest Ripening

**DOI:** 10.3390/foods12071509

**Published:** 2023-04-03

**Authors:** Yi Chen, Xiaomin Hu, Qingke Shi, Yue Lu, Jing Yan, Ding-Tao Wu, Wen Qin

**Affiliations:** 1 College of Food Science, Sichuan Agricultural University, Ya’an 625014, China; 2020218003@stu.sicau.edu.cn (Y.C.); huxiaomin@stu.sicau.edu.cn (X.H.); sicaui@163.com (Q.S.); luyue021212@163.com (Y.L.); yanjing@sicau.edu.cn (J.Y.); 2 Institute of Food Processing and Safety, Sichuan Agricultural University, Ya’an 625014, China; 3 Institute for Advanced Study, Chengdu University, Chengdu 610106, China

**Keywords:** kiwifruit, phenolic compounds, HPLC analysis, antioxidant capacity, correlation analysis

## Abstract

Kiwifruit is very popular for its unique flavor and nutritional value, and for its potential health benefits, which are closely related to its richness in a variety of natural antioxidant substances, in which polyphenolics play a non-negligible role. This study investigated changes in the fruit quality, phenolic compounds, and antioxidant potential of Chinese red-fleshed kiwifruit “Hongshi No. 2” during postharvest ripening at room temperature (20 ± 1 °C). Results showed that the weight loss rate slowly increased, the firmness rapidly decreased, and the soluble solid concentration gradually increased during the postharvest ripening of red-flesh kiwifruit. In addition, the total phenolic (TPC), total flavonoid (TFC), and total proanthocyanidin (TPAC) contents gradually increased during postharvest ripening. The most abundant phenolic compounds in kiwifruit throughout postharvest ripening were catechin (CC), proanthocyanidin B1 (PB1), and proanthocyanidin B2 (PB2). Furthermore, the methanolic extracts of red-flesh kiwifruit exhibited remarkable antioxidant activities throughout postharvest ripening stages. Indeed, some phenolic compounds showed good correlations with antioxidant activities; for instance, chlorogenic acid (CHL) showed a significantly positive correlation with ferric reducing antioxidant power (FRAP), and isoquercitrin (IS) showed a significantly negative correlation with DPPH free radical scavenging ability. The findings from this study are beneficial to better understanding the quality profile of red-flesh kiwifruit “Hongshi No. 2” during postharvest ripening.

## 1. Introduction

Kiwifruit (*Actinidia*), is subordinated to the genus *Actinidia* in the family *Actinidiaceae*, and has been domesticated and cultivated by humans for more than 100 years, since the beginning of the 20th century [1]. Kiwifruit is popular for its unique nutritional value, medicinal value, and high economic value. Rich in many functional components, kiwifruit has both edible value and certain health functions [2]. Not only does it contain a variety of essential functional components such as polyphenols, amino acids, vitamins, and minerals [3], but long-term consumption of kiwifruit can effectively replenish the body’s vitamins, strengthen the immune system, relieve skin inflammation, reduce serum cholesterol levels, and induce anti-aging, anti-viral, anti-radiation, anti-tumor, and cardiovascular and cerebrovascular disease prevention effects [4]. Studies have shown that these benefits are closely related to its richness in several natural antioxidant substances, especially the kiwifruit polyphenols [5,6]. For instance, Kurakane et al. [7] suggested that the polyphenol fraction of *Actinidia arguta* (AP) had antidiabetic effects, and isoquercitrin derived from AP might be useful in preventing type 2 diabetes mellitus. In addition, Xie et al. [8] found that phenolics from whole golden kiwifruit and fruit pomace possessed prebiotic potentials. Bursal et al. [9] found that the consumption of kiwifruit had beneficial effects due to its antioxidant properties. Blaszczak et al. [10] revealed that polyphenols derived from kiwi-berries could prevent the formation of advanced glycation end products (AGEs).

Phenolic components are important secondary metabolites that may directly affect the color, texture, hardness, and flavor of fruits [11,12]. They are an indispensable component of the human diet [13], and their content and activity are important indicators of fruit nutritional values [14]. Many studies on the phenolic compounds and antioxidant activities of kiwifruit have been reported. For example, Sharma et al. [15] analyzed and evaluated the quality changes of several cultivars of kiwifruit under ambient storage: ‘Hayward’, ‘Allison’, ‘Abbott’, ‘Bruno’, and ‘Monty’. It was found that ‘Hayward’ and ‘Allison’ contained higher phenolic content, and exhibited higher firmness values and lower weight loss rate (WLR) and decay loss. For the composition of phenolic substances in kiwifruit, Kim et al. [16] found that the major phenolic substances in hardy kiwi were epicatechin, procyanidin B type, procyanidin trimers, catechin, chlorogenic acid, and isoquercetin. Liu et al. [17] stated that chlorogenic acid, p-coumaric acid, and ferulic acid were the key polyphenols in red-fleshed kiwifruit. Most red-fleshed kiwifruit contains high levels of total phenolics and anthocyanins, besides superoxide dismutase (SOD), catalase (CAT) and peroxidase (POD) activities. In addition, these fruits are generally stronger as antioxidants than green-fruited kiwifruit, according to ABTS, DPPH, and FRAP criteria. Jiao et al. [18] studied the richness of flavonoids and phenolic acids in kiwifruit pulp. Epicatechin is the main phenolic compound that contributes to the antioxidant capacity. The phenolic content and antioxidant activity of Sungold and Sweetgreen varieties (pulp and rind) were significantly higher compared to the commercial variety, Hayward. However, most of these reported studies used mature fruits of varieties such as Hayward kiwifruit, and very limited studies were conducted on red-fleshed kiwifruit. In addition, the changes in fruit quality, phenolic accumulation, and antioxidant activity of red-flesh kiwifruit during postharvest ripening are rarely reported.

The Chinese kiwifruit “Hongshi No. 2” is a new hybrid red-flesh variety of “Hongyang” × “SF0612M”. The fruit is oval-shaped. with greenish-brown skin and yellowish-green flesh, with red and greenish-yellow patterns on the cross-section of the fruit [19]. It is very sweet, with a few short hairs evenly distributed on the skin surface. Compared with the main cultivar “Hongyang”, the new variety “Hongshi No. 2” has no hollow fruit, stronger red color, brighter color, better taste, and stronger resistance, which has reached a certain planting area and has greater commercial prospects [20]. However, research on the functional substances of this variety is still very limited. Li et al. [21] found that “Hongshi No. 2” kiwifruit has higher phenolics content and better antioxidant capacities than those of other varieties. However, information on the changes in its quality and bioactive substances during postharvest softening at room temperature is very limited, especially for the bioactive substances presented in the kiwifruit at the edible stage. Therefore, it is necessary to reveal the changes in the quality profile of red-flesh kiwifruit “Hongshi No. 2” during postharvest ripening, which is conducive to improving the quality of kiwifruit during postharvest storage.

Therefore, the objective of this study was to analyze the dynamic changes in fruit quality, phenolic compounds, and antioxidant activity of the red-fleshed kiwifruit “Hongshi No. 2” during postharvest ripening.

## 2. Materials and Methods

### 2.1. Material

**Figure 1:**
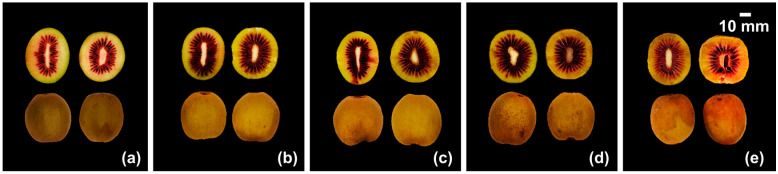
Morphological characteristics of kiwifruit “Hongshi No. 2” at different stages. Pictures (**a**–**e**) are the horizontal section and complete appearance of kiwifruits at different stages a, b, c, d, and e, respectively.

The *Actinidia chinensis cv.* “Hongshi No. 2” were planted in Deyang planting base in Sichuan Province (GPS coordinate 104°9′17″ E, 31°23′47″ N). They were harvested on 26 September 2020, when the concentration of soluble solids reached 7–9%, and were stored at room temperature (20 ± 1 °C). The firmness was estimated every 2 days. The samples were divided into five groups according to 5 post-ripening stages, according to the hardness and shape of the fruit. The division principle is shown in Table 1, and the morphological characteristics of “Hongshi No. 2” at different post-ripening stages are given in Figure 1. About 2 kg of samples was randomly selected at every stage for further analysis. These samples were flaked off and sliced, frozen with liquid nitrogen and put away at −80 °C for quality determination.

### 2.2. Determination of Fruit Quality

#### 2.2.1. Weight Loss Rate (WLR)

The evaluation of WLR referred to the study by Lu [22]. All fresh kiwifruit were weighed on an electronic balance to record the total mass of fruit every 2 days, and the average fruit weight was calculated. Fruit WLR was calculated using the following formula, expressed in percentage content (%).

$$WLR\%=\frac{A_{1}-A_{2}}{A_{1}}\times 100\%$$

where A_1_ represented the average weight of the fruit on the first day and A_2_ represented the average weight of the fruit on the day of post-ripening stages.

#### 2.2.2. Firmness

The firmness was measured based on Maarten LATM’s method, with appropriate modifications [23]. Six kiwifruits at each stage were randomly selected. Each fruit was peeled at four equatorial points, with a peel area of approximately 10 × 10 mm for hardness determination using a P/5 probe. The operating parameters were as follows: initial test speed of 10 mm·s^−1^; measurement velocity of 2 mm·s^−1^ and trigger strength of 5.0 g. Firmness results are expressed in Newton (N).

#### 2.2.3. Soluble Solid Concentration (SSC)

About 50 g of fresh kiwifruit at each stage was collected, ground evenly using a blender and filtered with four layers of gauze, and the clarified filtrate was collected. SSC was determined with a handheld digital refract orometer. The products were manifested as percentage content (%).

#### 2.2.4. Vitamin C (V_C_)

The determination of V_C_ was as described in previous research [24]. Briefly, 2.0 g of fresh samples was uniformized in pre-chilled trichloroacetic acid (TCA, 5%, *w*/*v*) on ice, then centrifuged at 4000× *g* at 4 °C for 10 min and the volume adjusted to 50 mL. TCA was used as blank control. A Varioskan Flash Multimode Reader was manipulated to take stock of the absorbance at 534 nm. A standard curve of V_C_ was used for calibration. The results were expressed as mg V_C_ per gram weight of fresh kiwifruit (mg·g^−1^).

### 2.3. Extraction and Analysis of Phenolic Compounds

#### 2.3.1. Extraction Process

The extraction of polyphenolics was followed by Li [21] with some modifications. Lyophilized kiwifruit powder (1.0 g) was stirred with acidified methanol solvent (0.1% HCl, *v*/*v*). Ultrasound-assisted extraction (50 kHz, 480 W) was performed twice at room temperature, and the solvent was halved for the second extraction with 60 min each time. Following centrifugation (5000× *g*, 15 min), the supernatant solution was pooled and concentrated at 45 °C with a rotary evaporator, and then the volume was adjusted to 25 mL with methanol for subsequent determination.

#### 2.3.2. Total Phenolic Content (TPC)

TPC was judged with the Folin–Ciocalteu procedure, which was explained by Everett [25] with slight modifications. In brief, 100 μL of kiwifruit extract dilution or gallic acid standard solution was added to 500 uL of forintanol reagent. The mixture was then reacted in the dark at room temperature (25 °C) with the addition of 500 μL Na_2_CO_3_ (20%, *w*/*v*) over 3 min. Finally, the reaction was carried out in the dark for 30 min at room temperature (25 °C). The absorbance of the sample was identified at 765 nm with a microplate reader, and methanol was used as the blank. TPC values were calculated by regression curves fixed from gallic acid standards (0, 0.02, 0.04, 0.08, 0.12, 0.16, and 0.20 mg·L^−1^). The outcome was presented in mg of gallic acid equivalents per gram weight of dry kiwifruit powder (mg GAE·g^−1^).

#### 2.3.3. Total Flavonoid Content (TFC)

The TFC was determined by Lin [26] with partial modifications. A total of 30 μL of 5% NaNO_2_ solution (*w*/*v*) was added to 100 μL of kiwifruit extract or rutin standard solution. After 6 min of reaction, 30 μL of 10% Al(NO_3_)_3_ (*w*/*v*) was added. Subsequently, 400 μL of 4% NaOH (*w/v*) was added and the reaction was carried out for 25 min at room temperature and protected from light. The absorbance of the sample was measured at 510 nm, and methanol was applied as the blank. TFC values were finished by a linear fitting equation fixed from rutin standards (0, 0.1, 0.2, 0.3, 0.4, 0.5, 0.6, 0.8 and 1.0 mg·mL^−1^). The outcome emerged as mg of rutin equivalents per gram weight of dry kiwifruit powder (mg RE·g^−1^).

#### 2.3.4. Total Proanthocyanidin Content (TPAC)

The TPAC was determined with the Vanillin sulfuric acid method [27]. A total of 750 μL of vanillin methanol solution was added to 750 μL of sulfuric acid methanol solution, and then 300 μL of kiwifruit extract or catechin standard solution was immediately added. Finally, the mixture was incubated at 30 °C for 20 min. The absorbance of the sample was scaled at 500 nm, with methanol as the blank. TFC values were judged by a linear regression curve provided from a series of catechin standards (0, 0.2, 0.4, 0.8, 1.0, 1.4, and 1.8 mg·mL^−1^). The outcome was provided in mg of catechin equivalents per gram weight of dry kiwifruit powder (mg CE·g^−1^).

#### 2.3.5. HPLC Analysis

Qualitative and quantitative interpretations of individual phenolic compound were carried out as in Li [21]. The content of the individual phenolic compound emerged as micrograms per gram weight of dry kiwifruit powder (μg·g^−1^). HPLC analysis was performed using the column Phenomenex Gemini C18 110A (150 mm × 4.6 mm, 5 µm). The mobile phase was 0.5% acetic acid (A)-acetonitrile (B). The flow rate and injection volume were 0.8 mL/min and 20 μL, respectively. The detection wavelength was 280 nm. Eight standard substances were selected, including gallic acid (GA), protocatechuic acid (PA), procyanidin B1 (PB1), (+)-catechin (CE), chlorogenic acid (CA), procyanidin B2 (PB2), (−)-epicatechin (EC), and isoquercitrin (IS).

### 2.4. Determination of In Vitro Antioxidant Activity

The antioxidant activity of kiwifruits at each storage time was detected by the free radical scavenging capacity (DPPH and ABTS) and FRAP. For DPPH scavenging capacity [28,29], the methanolic extract was first diluted, and 100 μL of the sample solution was mixed with 800 μL of 0.35 mM DPPH, then incubated at 37 °C for 30 min. The absorbance values were obtained at 517 nm. For ABTS scavenging capacity [29,30], 7 mM ABTS solution was mixed with 2.45 mM potassium persulfate solution in equal proportions and then reacted for more than 16 h under protection from light to obtain the ABTS working solution. Then, 200 μL of ABTS working solution was added to 20 μL of samples at different concentrations, and the absorbance was measured at 734 nm after 10 min reaction at 37 °C. IC_50_ values were summarized in the concentration of the kiwifruit sample solution (mg·mL^−1^). For FRAP capacity [31], FRAP working solution was obtained by mixing 300 mM acetate buffer solution, 10 mM TPTZ hydrochloric acid solution, and 20 mM ferric chloride solution in proportion, and preheated to 37 °C before use. Then, 100 μL of different concentrations of samples were mixed with 3 mL of working solution at 1 min intervals and the absorbance values were measured at 593 nm after a dark reaction at 37 °C for 4 min. The FRAP activity of kiwifruit was displayed as Trolox equivalents per gram of dry weight of kiwifruit (μmol·g^−1^).

### 2.5. Statistical Analysis

Data analysis was accomplished with IBM SPSS Statistics 27. The results were given by the mean ± standard deviation (*n* ≥ 3). ANOVA was used to compare statistically with Duncan’s multi-range test. The disparity was found to be statistically significant at *p* < 0.05. Pearson’s relevance coefficients were finished with Origin 2023 software.

## 3. Results and Discussion

### 3.1. Changes in Fruit Quality

The variations in WLR, firmness, SSC, and V_C_ of “Hongshi No. 2” kiwifruit during postharvest ripening at room temperature are presented in Table 2. There was a slow increase in WLR from stages A to D, by 4.70%, and then a sharp increased from D to E, by 9.20%. An increment in the WLR of kiwifruit during postharvest ripening was commonly observed; for example, the WLR of red-flesh kiwifruit was 23.79% during storage at room temperature [32], which was much higher than that in this study, probably due to the different kiwi varieties. Firmness is extensively emphasized to characterize the postharvest quality of kiwifruit [33]. With the elongation of the conservation time, the firmness of kiwifruit showed a significant decrease from stage A to D, while only a slight decrease was observed from stages D to E. Notably, the fastest decrease in firmness was observed from stages B to C. This phenomenon might be because kiwifruit is a climacteric fruit that tends to soften when ethylene reaches a certain level during the post-ripening process [34]. The SSC in kiwifruit increased first, and then slightly reduced, with a maximum value at edible stage (D stage) of 15.68%, which was in accord with a previous study [35]. The increase in SSC was mainly due to the starch degradation during post-ripening, which accelerated the accumulation of soluble solids concentration [36]. The V_C_ content decreased along with the storage time, and stayed stable after the fruit was completely softened, and the V_C_ content in stage E was similar to that in stages C and D. Similar results were also found in other studies [37]. Kiwifruit is known as “the king of V_C_” because of its rich V_C_ content [38]. The V_C_ content in “Hongshi No. 2” was 3.13 mg·g^−1^ at the immature stage (C stage), which was much higher than other red-flesh kiwi varieties with V_C_ content of 0.644 mg·g^−1^ [39], indicating that this variety might be more nutritional than other varieties.

### 3.2. Changes in Phenolic Profiles

The changes in TPC, TFC, and TPAC are shown in Table 2. An increasing trend in TPC was detected during postharvest ripening stages, increasing from 7.89 mg GAE·g^−1^ (stage A) to 10.00 mg GAE·g^−1^ (stage E). The total phenolic content of kiwifruit during storage is reported to be related to storage conditions; when stored at a low temperature, it may increase with the storage time [40]. However, the TPC of kiwifruit may also decrease during storage at room temperature, probably due to different preservation methods [41]. The highest TFC was observed in the edible phase (stage D), with 10.39 ± 0.08 mg RE·g^−1^ among all ripening stages. In most previous studies, the TFC of kiwifruit decreased during postharvest storage, and with some preservation, treatment could inhibit the reduction of total flavonoids [42]. However, several kiwi varieties showed an opposite tendency; for example, the total phenolic content and total flavonoid content in ‘haegeum’ kiwifruit increased during storage stages [43]. This phenomenon might because of the spontaneous up-regulation of phenolic and flavonoid contents in kiwifruit to resist unfavorable conditions such as spoilage and deterioration in the phenol–propane metabolic pathway [44]. Proanthocyanidins are metabolites in the flavonoid metabolic pathway and are abundant in red-fleshed kiwifruit [45]. In this study, proanthocyanidins content increased from 11.84 mg CE·g^−1^ to 14.56 mg CE·g^−1^ during post-ripening stages. It was evident in Figure 1 that the color of the red heart part of kiwifruit became darker with the extension of storage time. Similar results were also found in the “Hongyang” kiwi variety, in which anthocyanin content increased during low-temperature storage [46].

**Figure 2:**
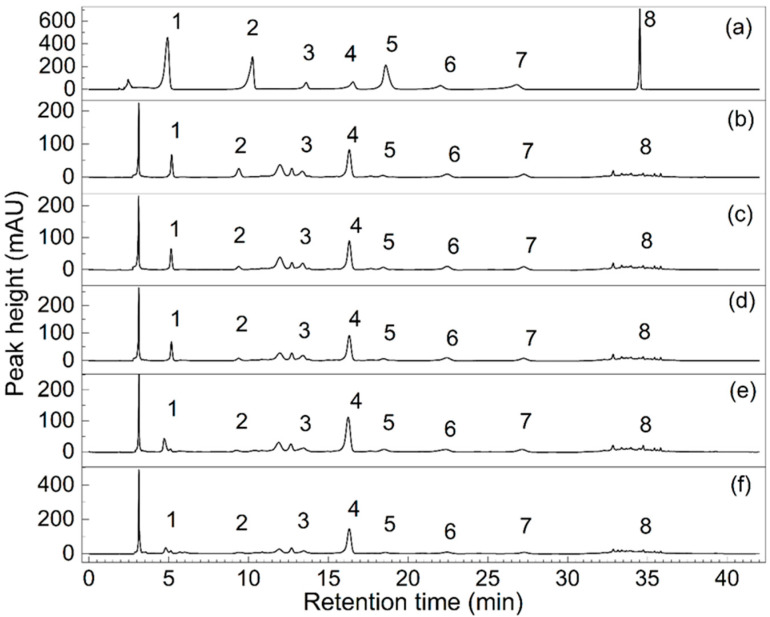
Chromatograms of phenolic standards and kiwifruit samples at different stages. (**a**) Represents the chromatogram of the mixed standard, and (**b**–**f**) are the chromatograms presented for the five stages: A, B, C, D, and E, respectively, where 1 is gallic acid, 2 is protocatechuic acid, 3 is procyanidin B1,4 is (+)-catechin, 5 is chlorogenic acid, 6 is procyanidin B2, 7 is (−)-epicatechin, and 8 is isoquercitrin.

Figure 2 showed the chromatograms of phenolic standards and kiwifruit samples. The regression equations of the eight standards were as follows: GA: y = 0.7965x − 0.1176, R^2^ = 0.9991, PA: y = 0.5422x + 0.0195, R^2^ = 0.9990, PB1: y = 0.1463x + 0.0219, R^2^ = 0.9999, CE: y = 0.2637x − 0.3900, R^2^ = 0.9993, CA: y = 0.6606x − 0.1152, R^2^ = 0.9994, PB2: y = 0.1546x − 0.0062, R^2^ = 0.9995, EC: y = 0.2315x + 0.0271, R^2^ = 0.9990, IS: y = 0.3663x + 0.0247, and R^2^ = 1.0000. The contents of the eight phenolic compounds detected at different stages are shown in Table 3. The GA content, remarkably, weakened from 136.23 μg·g^−1^ (stage A) to 90.34 μg·g^−1^ (stage E) (*p* < 0.05). The EC content showed an overall decreasing trend from 282.23 μg·g^−1^ (stage A) to 230.18 μg·g^−1^ (stage C), followed by a fluctuation in stages D and E. The PA content obviously increased from 23.80 μg·g^−1^ (stage A) to 92.72 μg·g^−1^ (stage E). Similar trends were found in PB1 and PB2; both showed the minimum content at the edible stage (stage D) with 414.51 μg·g^−1^ and 346.46 μg·g^−1^, respectively, and then significantly increased at the decay stage (stage E). IS content increased from 24.09 μg·g^−1^ (stage A) to 41.65 μg·g^−1^ (stage E). CE was the most abundant phenolic compound detected, and it enhanced remarkably with storage time (*p* < 0.05). Unlike other phenolic compounds, the content of CHL peaked at the edible stage (stage D) at 92.23 μg·g^−1^. Other phenolic compounds in kiwifruit varieties were mentioned in the study and found in comparison under similar chromatographic conditions [21,47]. The content of phenolic compounds in the “Hongshi No. 2” kiwi variety was higher than most in other varieties, for instance *A. deliciosa cv.* Hayward, *A. chinensis cv.* Hongyang, etc. [21]. Additionally, from the chromatogram, some absorption peaks with large peak areas did not fit corresponding standards, suggesting that there might be more diverse phenolic compounds in the methanolic extract of kiwifruit “Hongshi No. 2” which require further identification.

### 3.3. In Vitro Antioxidant Capacities

**Figure 3:**
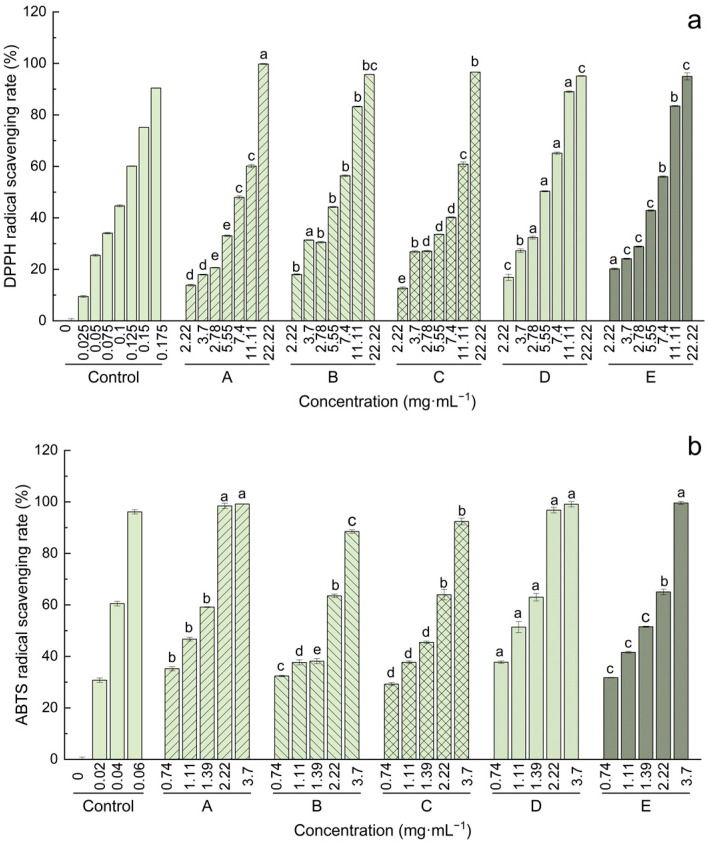
DPPH (**a**) and ABTS (**b**) free radical scavenging abilities of methanolic extracts of kiwifruit at different stages. Different letters (a–e) at the same concentration indicate significant differences in antioxidant potentials of samples during different post-softening stages.

As shown in Figure 3, the methanolic extracts of kiwifruit at different stages notably exhibited DPPH and ABTS radical scavenging abilities in a dose-dependent manner. The IC_50_ values of DPPH and ABTS scavenging capacities and FRAP capacity are displayed in Table 3. More specifically, the DPPH radical scavenging capacity gradually increased with the increase in storage times. The highest DPPH radical scavenging capacity was found in the edible stage (stage D), while lower activity was shown at the unripe stage (stages A, B, and C). In addition, the higher ABTS radical scavenging capacity was found in the edible stage (stage D), while lower activity was detected in the early stages (stages B and C), mainly due to the accumulation of total phenolics. Interestingly, higher ABTS radical scavenging capacity was also observed in stage A, which might be due the combined effect of Vc and phenolics. FRAP showed an overall increasing trend and peaked at the edible stage (stage D) with 116.68 ± 1.32 μmol·g^−1^, and then decreased after over ripening. These results indicated that the cumulative or synergistic effects of secondary metabolites of kiwifruit might increase the antioxidant activity of the fruit [48].

### 3.4. Correlation Analysis

**Figure 4:**
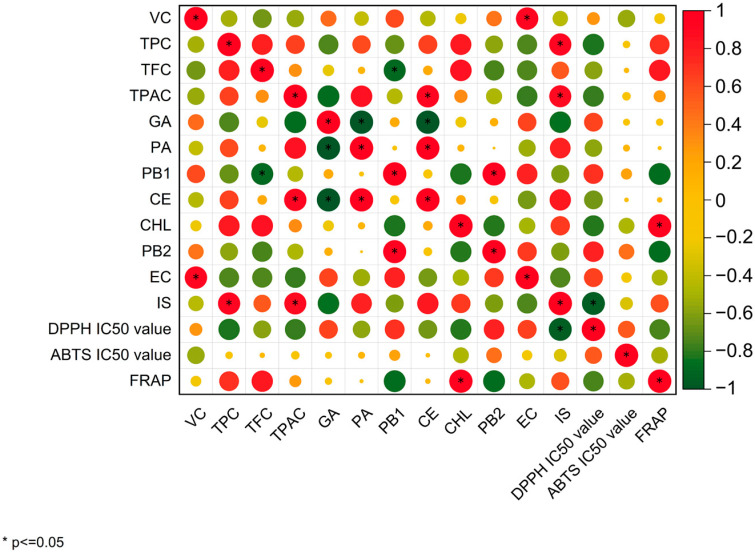
Pearson correlation matrix among Vc, phenolics, and antioxidant activity. The correlation coefficients are proportional to the circular size and color intensity. The positive correlation is displayed in orange, and negative in green. * shows significant correlation at *p* < 0.05.

Figure 4 showed the Pearson’s correlation heat map of TPC, TFC, TPAC, individual phenolic compounds, and antioxidant capacities of “Hongshi No. 2” kiwifruit. Results indicated that TPC, TFC, and TPAC were the major contributors to the DPPH radical scavenging ability, and TPC and TFC were also the major contributors to the FRAP capacity. However, Vc and CHL might be the major contributors to the ABTS radical scavenging ability. Furthermore, CHL also showed a positive correlation with FRAP and a negative correlation with DPPH radical scavenging ability, which was consistent with a previous study in which the higher the CHL content during coffee brewing, the higher the antioxidant activity in the coffee drink [49]. In addition, IS showed a negative correlation with DPPH free radical scavenging ability, and the result was also reported in a previous study [50]. Nevertheless, it was speculated that the changes in the antioxidant activity of kiwifruit during post-ripening were not caused by a single phenolic compound, but by the coordination of a variety of phenolic compounds.

## 4. Conclusions

In this study, the quality profile of red-fleshed kiwifruit “Hongshi No. 2” during postharvest ripening was revealed according to the dynamic changes in fruit quality, phenolic compounds, and antioxidant activity. Results showed that the weight loss rate of kiwifruit slowly increased, and the soluble solid concentration gradually increased. In addition, the total phenolic (TPC), total flavonoid (TFC) and total proanthocyanidin (TPAC) contents gradually increased. Furthermore, the methanolic extracts of red-flesh kiwifruit exhibited remarkable antioxidant activities throughout postharvest ripening stages. In fact, both TPC and TFC were the major contributors to the DPPH radical scavenging ability and FRAP capacity. Additionally, some phenolic compounds, such as CHL and IS, showed good correlations with antioxidant activities. The findings from this study can provide theoretical bases for controlling the quality of red-fleshed kiwifruit “Hongshi No. 2” during postharvest storage.

## Figures and Tables

**Table 1:** The division principle of 5 post-ripening stages.

Post-Ripening Stages	Days of Storage (d)	Morphological Description
A	0–2	unripe, very hard
B	4–6	unripe, hard
C	8–10	unripe, semi-soft
D	12–14	eating-ripe, soft
E	16–18	overripe, very soft

**Table 2:** Changes in fruit qualities and bioactive ingredients of kiwifruit “Hongshi No. 2” during postharvest ripening.

Stages	WLR (%)	Firmness (N)	SSC (%)	V_C_ (mg·g^−1^)	TPC (mg GAE·g^−1^)	TFC (mg RE·g^−1^)	TPAC (mg CE·g^−1^)
A	0.00 ± 0.14 ^e^	18.15 ± 0.82 ^a^	9.67 ± 0.27 ^d^	3.13 ± 0.08 ^a^	7.89 ± 0.29 ^c^	5.02 ± 0.64 ^d^	11.84 ± 0.45 ^c^
B	1.74 ± 0.12 ^d^	13.52 ± 2.49 ^b^	12.14 ± 0.19 ^c^	2.66 ± 0.18 ^b^	9.01 ± 0.07 ^b^	8.28 ± 0.06 ^bc^	11.26 ± 0.59 ^c^
C	3.10 ± 0.18 ^c^	5.46 ± 1.51 ^c^	14.08 ± 0.37 ^b^	2.28 ± 0.15 ^c^	8.74 ± 0.03 ^b^	8.65 ± 0.20 ^b^	12.90 ± 0.71 ^b^
D	4.70 ± 0.22 ^b^	0.86 ± 0.83 ^d^	15.68 ± 0.19 ^a^	2.65 ± 0.15 ^b^	10.25 ± 0.09 ^a^	10.39 ± 0.08 ^a^	13.11 ± 0.19 ^b^
E	9.20 ± 0.02 ^a^	0.27 ± 0.07 ^d^	14.47 ± 0.17 ^b^	2.42 ± 0.03 ^bc^	10.00 ± 0.11 ^a^	7.87 ± 0.04 ^c^	14.56 ± 0.40 ^a^

Values are means ± standard deviation (*n* = 3). Different letters in the same column indicate significant differences (*p* < 0.05).

**Table 3:** Changes in phenolic compounds and antioxidant potentials of kiwifruit at diverse post-ripening stages.

Phenolic Compounds (μg·g^−1^) /Antioxidant Activity	Stages				
	A	B	C	D	E
Gallic acid	136.23 ± 0.80 ^a^	129.74 ± 0.85 ^b^	129.80 ± 0.90 ^b^	120.53 ± 0.16 ^c^	90.34 ± 0.16 ^d^
Protocatechuic acid	23.80 ± 0.19 ^e^	27.66 ± 0.18 ^d^	26.35 ± 1.00 ^c^	37.30 ± 0.12 ^b^	92.72 ± 0.23 ^a^
Procyanidin B1	609.17 ± 0.66 ^a^	568.02 ± 0.06 ^b^	463.55 ± 0.38 ^d^	414.51 ± 0.07 ^e^	530.55 ± 0.96 ^c^
(+)-catechin	994.11 ± 0.92 ^e^	998.83 ± 1.88 ^d^	1087.20 ± 0.54 ^c^	1195.54 ± 0.13 ^b^	1738.73 ± 0.58 ^a^
Chlorogenic acid	43.14 ± 0.15 ^d^	54.29 ± 0.05 ^c^	54.55 ± 0.50 ^c^	92.23 ± 0.33 ^a^	57.72 ± 1.05 ^b^
Procyanidin B2	469.42 ± 0.9 ^b^	481.27 ± 0.71 ^a^	389.58 ± 0.39 ^d^	346.46 ± 0.10 ^e^	436.83 ± 0.84 ^c^
(−)-epicatechin	282.23 ± 0.70 ^a^	261.68 ± 0.98 ^b^	230.18 ± 0.37 ^d^	237.47 ± 0.10 ^c^	229.01 ± 0.48 ^d^
Isoquercitrin	24.09 ± 0.72 ^d^	24.52 ± 0.22 ^d^	28.48 ± 0.43 ^c^	39.79 ± 0.75 ^b^	41.65 ± 0.36 ^a^
DPPH (mg·mL^−1^)	10.25 ± 0.04 ^b^	11.11 ± 0.01 ^a^	9.44 ± 0.03 ^c^	5.87 ± 0.02 ^e^	7.07 ± 0.02 ^d^
ABTS (mg·mL^−1^)	1.17 ± 0.06 ^c^	1.76 ± 0.04 ^a^	1.61 ± 0.06 ^b^	1.07 ± 0.01 ^c^	1.51 ± 0.06 ^b^
FRAP (μmol·g^−1^)	91.03 ± 0.37 ^d^	95.18 ± 0.70 ^c^	98.20 ± 1.75 ^b^	116.68 ± 1.32 ^a^	95.75 ± 1.77 ^bc^

Values are means ± standard deviation (*n* = 3). Different letters in the same row indicate significant differences (*p* < 0.05).

## Data Availability

Data are contained within the article.
